# Redox System and Oxidative Stress-Targeted Therapeutic Approaches in Bladder Cancer

**DOI:** 10.3390/antiox13030287

**Published:** 2024-02-26

**Authors:** George J. Dugbartey, Sydney Relouw, Liam McFarlane, Alp Sener

**Affiliations:** 1 Department of Surgery, Division of Urology, London Health Sciences Centre, University of Western Ontario, London, ON N6A 5A5, Canada; 2 Matthew Mailing Center for Translational Transplant Studies, London Health Sciences Centre, Western University, London, ON N6A 5A5, Canada; 3 Multi-Organ Transplant Program, London Health Sciences Centre, London, ON N6A 5A5, Canada; 4 Department of Pharmacology and Toxicology, School of Pharmacy, College of Health Sciences, University of Ghana, Legon, Accra P.O. Box LG43, Ghana; 5 Department of Physiology & Pharmacology, Accra College of Medicine, Accra P.O. Box CT 9828, Ghana; 6 Department of Microbiology and Immunology, University of Western Ontario, London, ON N6A 3K7, Canada

**Keywords:** bladder cancer (BCa), redox, reactive oxygen species (ROS), oxidative stress, antioxidant therapy

## Abstract

Bladder cancer (BCa) is the most common genitourinary malignancy, with a high global incidence and recurrence rate that is paired with an increasing caregiver burden and higher financial cost, in addition to increasing morbidity and mortality worldwide. Histologically, BCa is categorized into non-muscle invasive, muscle invasive, and metastatic BCa, on the basis of which the therapeutic strategy is determined. Despite all innovations and recent advances in BCa research, conventional therapies such as chemotherapy, immunotherapy, radiotherapy, and surgery fall short in the complete management of this important malignancy. Besides this worrying trend, the molecular basis of BCa development also remains poorly understood. Burgeoning evidence from experimental and clinical studies suggests that oxidative stress resulting from an imbalance between reactive oxygen species (ROS) generation and the body’s antioxidant production plays an integral role in BCa development and progression. Hence, ROS-induced oxidative stress-related pathways are currently under investigation as potential therapeutic targets of BCa. This review focuses on our current understanding regarding ROS-associated pathways in BCa pathogenesis and progression, as well as on antioxidants as potential adjuvants to conventional BCa therapy.

## 1. Introduction

According to data from Global Cancer Statistics, bladder cancer (BCa) is the 10th most common form of cancer globally, with over 573,000 new cases in 2020 [1]. BCa is also the 13th most deadly cancer, causing 213,000 deaths worldwide in the year 2020 [1]. The prevalence of BCa is so high that about 1 in 100 men and 1 in 400 women will develop bladder cancer at some point in their lifetime [2]. This indicates that the BCa prevalence is about four times higher in men than in women, with incident rates of 9.6 and 2.4 per 100,000, respectfully [1]. In fact, BCa is the sixth most common cancer and the ninth leading cause of cancer death in men [1]. BCa is especially common in developed nations, particularly in Southern and Western Europe and North America [1,3]. Despite being at lower risk, females who are diagnosed with BCa are more likely to be at an advanced stage and tend to be at a higher risk of recurrence, progression, and mortality [4]. Attempts to explain these gender-based disparities in BCa have been concentrated on differences in risk factor exposure, metabolic detoxification of carcinogens, hormones, and diagnostic evaluation of hematuria [4]. Irrespective of gender, risk factors for BCa development consist of age, cigarette smoking, environmental and occupational mutagen exposure, and genetic predisposition [3,5,6,7]. Apart from these factors, benzidine, 2-naphthylamine, and arsenic have been identified as major BCa-inducing mutagens [8]. As such, there has been a high historic prevalence of BCa among workers in the industries of textile dyes and rubber, as well as in populations with contaminated water supplies [8]. These mutagens share pro-oxidant properties and can induce severe oxidative DNA damage [9,10,11].

An overwhelming majority of bladder malignancies are urothelial carcinomas (about 90%), with the remaining cases consisting of mostly squamous cell carcinoma and adenocarcinoma [12]. The classification of BCa is contingent on both the stage and grade of the tumor. The tumor stage is assigned based on the depth of the bladder wall invasion and the presence of lymph nodes and distant tissue metastases [12]. The tumor grade is dependent on a histological analysis of the tumor tissue, where architectural and cytological atypia are assessed to determine if the tumor is a papillary urothelial neoplasm of low malignant potential or a low-grade, high-grade, or benign growth [6]. The current diagnostic approach for BCa typically employs a cystoscopy and urine cytology, followed by transurethral resection of the tumor to confirm the diagnosis and evaluate the severity. However, recent research has focused on the use of urine biomarkers as a non-invasive option [13,14]. About 75–85% of BCa patients have non-muscle invasive bladder cancer (NMIBC), of which there is an equal distribution of high- and low-grade malignancies [6,15]. Conversely, muscle invasive bladder cancer (MIBC) is almost exclusively high-grade and constitutes the remaining pathologies [6].

The current recommended treatment regimens for BCa rely heavily on the stage and grade of the tumor. For low-grade tumors staged as Ta (non-invasive papillary urothelial carcinoma), intravesical chemotherapy using gemcitabine or mitomycin is generally utilized [16]. Transurethral resection is utilized for high-grade Ta tumors, which is optimally followed by a 3-year course of intravesical immunotherapy using Bacillus Calmette–Guérin (BCG) vaccine, although intravesical chemotherapy is also commonly utilized [16,17]. For NMIBC staged as T1 (tumor invades lamina propria), repeated transurethral resections followed by BCG therapy are recommended for low-grade tumors, and radical cystectomy is considered for high-grade tumors [16]. The standard of care for MIBC typically involves neoadjuvant therapy, radical cystectomy, and local lymph node dissection, although bladder-sparing treatments such as partial cystectomy or chemoradiation can be utilized in specialized cases [5,18]. These treatment protocols have remained more or less unchanged for several decades [19]. Despite this, the utilization of BCG as the primary therapeutic option for the treatment of BCa presents several challenges. In recent years, a global shortage of BCG has limited its availability as a therapeutic option [20]. Additionally, 20–40% of patients receiving BCG therapy are either unresponsive or experience tumor recurrence [21]. Furthermore, no widely effective non-surgical treatment options for true BCG-unresponsive patients exist, although many proposed alternatives are currently being investigated [22,23]. There is an imperative need for effective novel therapies, including adjunctive therapies to improve patient outcomes, particularly when BCG therapy proves ineffective. An interesting avenue of research on possible therapeutic developments is concerned with oxidative stress regulation. This review discusses the contribution of oxidative stress to BCa development and progression and antioxidants as potential adjuncts in BCa therapy.

## 2. Redox Homeostasis

**Figure 1:**
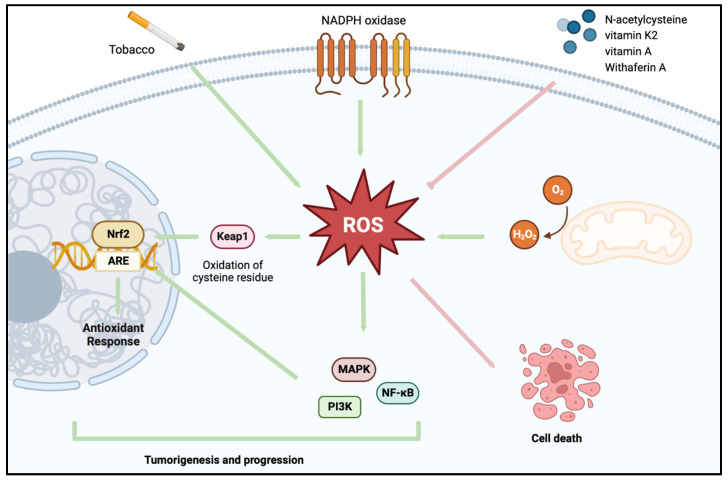
ROS-mediated signaling pathways involved in promoting and inhibiting BCa. Reactive oxygen species (ROS) are produced by endogenous (mitochondria and NADPH oxidase complexes) and exogenous (tobacco) sources, and mediate molecular pathways such as Nrf2, MAPK, NF-ĸB, and PI3K signaling pathways to promote tumorigenesis and progression. Antioxidants such as N-acetylcysteine, vitamin K2, vitamin A, and Withaferin A act as ROS scavengers, leading to death of cancer cells. Figure prepared with BioRender.

The maintenance of redox homeostasis is an essential process that is constantly occurring within healthy cells. Both endogenous and exogenous sources of oxidative stress, due to the overproduction of reactive oxygen species (ROS; destructive mediator of cell and tissue injury), are moderated through enzymatic and non-enzymatic cellular antioxidants. ROS are important redox signaling molecules that are involved in several pathophysiological pathways. They take the form of a superoxide anion (O_2_^−^), which can be transformed into hydrogen peroxide (H_2_O_2_) via superoxide dismutases, which can subsequently be transformed into a peroxyl radical (OH^-^), the most reactive ROS. Other ROS include singlet oxygen (^1^O_2_), ozone (O_3_), and organic hydroperoxides (ROOH). ROS are mainly produced by the mitochondria [24,25], namely, the electron transport chain, and NADPH-dependent oxidase complexes (Figure 1) [26,27], and also by the endoplasmic reticulum and peroxisomes.

At the physiological levels, ROS contribute to protein functions through reversible redox reactions involving processes such as metabolism, cell proliferation, differentiation, survival, and angiogenesis [26,28,29,30]. However, a shift in the redox system in favor of ROS occurs under pathological conditions, with several signaling pathways being implicated due to an overproduction of ROS, which induces damage to proteins, nucleic acids, lipids, membranes, and organelles and ultimately leads to apoptotic cell death [18,19,20,21,31]. In turn, oxidants are regulated by endogenous enzymes or cofactors known as antioxidants and form extensive defense systems against the damaging effects of ROS-induced oxidative stress. A well-known antioxidant defense system for maintaining redox homeostasis is the NF-E2-related factor 2-Kelch-like ECH-associated protein 1 (Nrf2-Keap1) system [32]. Under oxidizing conditions, Keap1 releases Nrf2, which undergoes nuclear translocation to upregulate antioxidant response elements (AREs) such as glutathione peroxidases, thioredoxins, peroxiredoxins, and superoxide dismutase [32,33].

### A Shift in the Redox System in BCa

ROS have been implemented as an important mediator of the initiation and promotion of carcinogenesis, as well as tumor progression [25,26]. Hence, ROS and associated pathways have been considered as a therapeutic target in cancer research. Increased production of ROS is observed in many known carcinogenetic pathways, particularly in conditions of chronic inflammation or exposure to exogenous stressors, such as cigarette smoke [34,35] (Figure 1). The activation of oncogenes has been demonstrated to drive aberrant ROS production, which in turn drives mitogenic signaling and, thus, the hyperproliferation of tumor cells [36]. The heightened metabolic activity of rapidly proliferating cells, coupled with the hypoxic tumor microenvironment, further contributes to ROS generation [35]. BCa patients exhibit heightened urinary levels of 8-iso-prostaglandin F2 α (8-iso-PGF2 α), a recognized biomarker of oxidative stress [37]. This is also true of several plasma biomarkers of oxidative stress such as malondialdehyde, thiobarbituric acid reactive substances, 3-nitrotyrosine, carbonyl groups, and thiol groups, with the latter two also correlating with the cancer stage and grade [34,35]. Collectively, this suggests an important role of ROS in the pathogenesis of BCa, with significantly lower intratumor activity and a downregulated expression of antioxidant enzymes, such as superoxide dismutase, glutathione peroxidase, and catalase, in tumor tissue compared to healthy tissue [38,39,40,41,42,43].

As illustrated in Figure 1, some of the signaling pathways associated with BCa transformation and progression include Nrf2/Keap1/ARE signaling, mitogen-activated protein kinase (MAPK), nuclear factor-kappa B (NF-ĸB), phosphatidylinositol-3-kinase (PI3K/Akt), and mammalian target of rapamycin (mTOR) pathways [31,44,45,46,47,48]. In addition to the endogenous sources of ROS production and as illustrated in Figure 1, oxidative stress leading to BCa can result from tobacco smoke [49], which accounts for 30–50% of all BCa cases [50]. This increased oxidative stress from both endogenous and exogenous sources of ROS causes DNA damage and protein dysregulation and results in increased mutations and hyper- and hypomethylation of tumor suppressors and oncogenes, respectively, within BCa cells. For example, mutations in the tumor suppressor p53 are present in over 50% of BCa patients, whereas mutations in the oncogene Ras are present in 30% of patients [51,52]. Oncogenic Ras mutations have been demonstrated to induce increased intracellular ROS generation and contribute to oxidative DNA damage [53]. In instances where mutations have also rendered p53 inactive, ROS generation and mutagenesis induced by oncogenic Ras are further exacerbated.

The proliferation of cancer cells leads to the production of increased levels of ROS due to increased metabolic demand and modified antioxidant defense systems [31]. In the early stages of tumor development, increased ROS generation can be attributed to the high proliferation rate of tumor cells, where the oxygen demand quickly exceeds what is supplied due to insufficient angiogenesis [44]. The resulting hypoxic tumor microenvironment attenuates electron flow through the electron transport chain, leading to mitochondrial ROS generation [54]. ROS have been shown to play a key role in the metabolic adaption to hypoxia by stabilizing hypoxia-inducible factors and upregulating related genes that are involved in angiogenesis [55]. Specifically, the expression of hypoxia-inducible factor-1α (HIF-1α) has been shown to be induced by ROS [56]. The downstream effects of hypoxia-inducible factors include the regulation cell metabolism, survival, motility, basement membrane integrity, and angiogenesis, favoring tumor cell adaptation to the microenvironment [44,57]. Upon immunohistochemical analysis of 93 patient tissue samples, Theodoropoulos et al. [58] observed that HIF-1α immunoreactivity was positively correlated with the tumor grade and was an indicator of poor overall and disease-free survival. Furthermore, when the expression of hypoxia-related genes was used by Lui et al. to assign hypoxia risk scores, high scores were reported to correlate with a high grade and advanced tumor stage in BCa patients [59]. This high hypoxia risk score was also demonstrated to be an independent prognosis predictor. However, these findings did not translate to low-grade BCa patients. This discrepancy suggests that the role of ROS in BCa may differ depending on BCa characterization.

Emerging pre-clinical and clinical studies have revealed a significant increase in the levels of oxidants and a marked decrease in the levels of antioxidants in serum and bladder tissue of animals and patients with bladder tumors compared to those without BCa [60,61]. Interestingly, Gesit et al. [60] investigated the relationship between tumor grade and oxidant and antioxidant levels in serum and found no correlation. Another study investigated levels of the antioxidant enzymes, paraoxonase, and arylesterase in the serum of BCa patients [62]. Once again, significantly higher oxidant levels and markedly reduced antioxidant levels were found in BCa patients compared to healthy controls. Contrary to the reports by Gecit et al., serum antioxidant levels demonstrated a positive correlation with tumor grade, tumor diameter, and recurrence. However, significance was only found for tumor diameter and recurrence [62]. The upregulation of oxidants and downregulation of antioxidants in BCa has also been supported by more recent findings [63,64,65]. Specifically, Sawicka et al. [64] investigated levels of lipid peroxidation and advanced oxidation protein products in terms of tumor stage and grade. Both products were significantly increased in BCa samples compared to healthy controls. Moreover, products of lipid peroxidation were slightly higher in the Ta stage of BCa compared to T1 and T2 and in G3 compared to G1 and G2. Advanced oxidation protein products, on the other hand, were highest in T1 and lowest in T2, and there was a slight negative correlation with increasing grade. A subsequent study grouped participants by grade and stage (TaG1 and T1G2) and found a positive correlation between the BCa classification and the level of oxidative stress [65]. Most recently, Zhang et al. [66] investigated the predictive role of oxidative stress-related genes in BCa and identified several genes, as well as reporting a positive correlation between the expression of these genes and the tumor stage and grade. Together, these findings suggest that oxidant and antioxidant levels contribute to BCa development and progression and can be used as potential therapeutic targets depending on the stage and grade of the BCa.

## 3. Utilizing Oxidative Stress-Based Strategies in the Treatment of BCa

As ROS have been shown to be an integral part of BCa cell adaptation, progression, and survival, these pathways have been investigated as potential therapeutic targets of BCa. The BCa tumor microenvironment exhibits heightened levels of ROS, and despite this environment conferring a survival advantage to tumor cells, it also provokes susceptibility to the triggering of ROS-induced cell death pathways. While the heightened antioxidant potential in tumor cells limits this, tumoral intracellular ROS still exceed those of healthy tissue. Some current effective cancer therapies such as chemotherapeutics, which exhibit the induction of intracellular ROS as an effector mechanism, benefit from this characteristic.

For example, in a recent study involving a subcutaneous xenograft murine model of BCa, Zhang and Li [67] investigated the effects of Nestin1, an angiogenesis marker, on cisplatin-treated BCa growth. By measuring tumor size, they found that Nestin1 overexpression partially reversed the anti-cancer effects of cisplatin, whereas the inhibition of Nestin1 potentiated its anti-cancer effects. In the context of non-small cell lung cancer, Wang et al. [68] demonstrated that Nestin1 overexpression stabilizes nuclear factor erythroid 2 (NFE2)-related factor 2 (Nrf2) by blocking Keap1-controlled ubiquitination-proteasomal degradation. As an important regulator of the cellular response to oxidative stress, promoting Nrf2 stability supports an elevated degree of cellular antioxidant responses [69]. This positive correlation between the expression of Nestin1 and protein levels of Nrf2 was also observed in human bladder cancer cells, and Nestin1 proved to be capable of mediating an antioxidant response to oxidative stress induced by cisplatin [67]. The increased susceptibility of BCa cells to cisplatin administration upon silencing the Nestin1 expression suggests that targeting antioxidant regulators may be an effective combination therapy strategy to mitigate the chemoresistance of BCa cells. Alternatively, the authors suggested that pharmaceutical promotion of Nrf2 transcriptional regulation could be an effective chemoprophylactic strategy against BCa and other malignancies by preventing the survival advantage that is conferred by ROS [67].

### 3.1. Eliminating the ROS-Facilitated Tumor Cell Survival Advantage

The strategy surrounding the use of antioxidants as chemoprophylactic pharmaceuticals has drawn a substantial amount of attention due to the important role that ROS play in the carcinogenesis of a wide range of cancer types. This strategy seeks to impair the survival advantage that is bestowed on tumor cells by oxidative stress in the early stages of carcinogenesis and tumor development [70]. Considering the important contribution of ROS-induced oxidative stress throughout BCa development and progression, exogenous antioxidants such as N-acetylcysteine, vitamin A, epigallocatechin-3-gallate (EGCG), and ellagic acid and its metabolite, urolithins, have been explored as potential BCa therapies (Table 1). In an in vitro model using a human BCa cell line, Supabphol et al. [71] investigated the anti-metastatic role of N-acetylcysteine and observed a dose-dependent effect of N-acetylcysteine on BCa cell viability, adhesiveness, migratory abilities, and invasiveness, with 50 mM of N-acetylcysteine abolishing almost all abilities. In a very recent study, mice who were fed a vitamin A-rich diet exhibited diminished early BCa progression compared to the untreated group [72]. Although not directly tested, vitamin A has potent antioxidant properties that were mentioned as a potential factor in the observed effect. Luo et al. [73] also investigated the effect of epigallocatechin gallate (EGCG; a polyphenol) on BCa in a subcutaneous xenograft murine model and found that EGCG significantly reduced tumor volume and weight compared to the control group. Lastly, ellagic acid and its urolithin metabolites were shown to exhibit antiproliferative effects on human bladder cancer cells. This effect was evidenced by decreased intracellular ROS and malondialdehyde, a result of the induction of increased SOD activity [74]. Clinically, the levels of antioxidants such as glutathione-s-transferase, reduced glutathione, superoxide dismutase, and glutathione reductase in the serum of 30 patients with a superficial bladder tumor were markedly reduced compared to 27 healthy volunteers. In the same clinical study, the authors observed a substantial increase in the level of serum xanthine oxidase (an oxidant indicator) in BCa patients in comparison with the control group [75]. These pre-clinical and clinical studies imply that antioxidants are protective against BCa and could serve as an effective adjunctive therapy.

While the results of these experimental and clinical studies appear to favor antioxidants as potential agents against oxidative damage in BCa development and progression by forming preventive or repair systems, emerging clinical studies also provide contrasting evidence. For example, in a multicenter, prospective, double-blinded, placebo-controlled, randomized clinical trial involving 270 NMIBC patients, supplementation with vitamin E and selenium (possessing nutritional and antioxidant properties) failed to reduce the risk of BCa recurrence, with no positive impact on overall patient survival [76]. In fact, the authors observed an increased risk of NMIBC following vitamin E administration, suggesting that its supplementation may be harmful to these patients [76]. In another clinical trial involving a cross-sectional study in which 90 BCa patients were recruited, significantly increased activities of antioxidant enzymes such as superoxide dismutase and catalase were associated with more invasive stages of BCa and with a higher progression potential [77]. This further suggests that antioxidants may have a dual role in BCa development and progression, and therefore, further investigations are required to provide a comprehensive understanding of the biology of antioxidants in the development and progression of BCa as well as other cancer types. Collectively, the findings from these studies suggest a potential for antioxidant use as chemoprophylactic agents or treatments in very early progressions of BCa, as well as a possible role in predicting and monitoring the trajectory of BCa. These findings also highlight the use of murine BCa models in the investigation of these therapies.

### 3.2. Utilizing ROS to Induce Tumor Cell Self-Destruction

The susceptibility of tumor cells to further increases in ROS makes pro-oxidants attractive candidates for the treatment of many malignancies, including BCa (Table 2) [78]. By inducing ROS generation or inhibiting endogenous antioxidant systems, pro-oxidants could be used to drive tumor cells to reach ROS-induced cell death thresholds. Unlike the strategy surrounding antioxidant use, which focuses on early intervention, pro-oxidants theoretically could have utility at any stage of tumor progression, since an elevated ROS level is ubiquitous. In BCa, the role of ROS in apoptotic pathways has been extensively studied. This induction of cell death can occur due to the direct damaging effects of ROS to proteins, nucleic acids, lipids, membranes, and organelles, or via ROS-initiated cell death signaling pathways. The direct induction of ROS is employed by many of the most effective cancer treatment strategies including radiotherapy and chemotherapies [78]. In fact, docetaxel and gemcitabine, the chemotherapeutic agents that have recently been shown to be effective against high-risk non-muscle invasive bladder cancer, have both been shown to induce ROS generation [79,80,81,82].

Choudhary and Wang [83] investigated the apoptotic role of oncogenic H-Ras in a human BCa cell line in conjunction with a histone deacetylase inhibitor. They found that oncogenic H-Ras-expressing BCa cells exhibited higher levels of ROS and increased susceptibility to treatment with H_2_O_2_ compared to the parental human BCa cell line. In a subsequent study, the same group [84] observed increased ROS-mediated cell death, while glutathione (the most abundant naturally occurring antioxidant) was downregulated by treatment with histone deacetylase inhibitor-treated H-Ras-expressing cells compared to the parental cell line. An investigation of ROS-induced tumor cell cytotoxicity has also been carried out in vivo. An example of this is a group’s investigation of the role of the solute carrier family 25 family member 21 (SLC25A21) in a subcutaneous xenograft murine model [85]. SLC25A21 is a key member of the SLC25 family that is involved in the oxidative stress response, inhibiting cell proliferation, migration, and invasion. They demonstrated that when SLC25A21 was overexpressed in subcutaneously inoculated BCa cells, the resultant tumor weight was significantly lower than in the control group, indicating an inhibitory role in BCa progression [85]. This effect was in part attributable to an increase in ROS levels, resulting in the induction of the mitochondrial apoptosis pathway [85].

Pro-oxidants that do not directly induce ROS generation instead target the activity of enzymatic antioxidants such as superoxide dismutase (SOD), glutathione peroxidase (GPx), or catalase (CAT), or non-enzymatic antioxidants such as thioredoxin (TRX) or the synthesis of molecular antioxidants such as glutathione (GSH) [78]. Glucose-6-phosphate dehydrogenase (G6PD) is a rate-limiting enzyme of the pentose–phosphate pathway, a major cellular antioxidant defense system. A study by Chen et al. [86] demonstrated the upregulation of G6PD in human bladder cancer tissue compared to healthy tissue and found higher expression levels to be a poor prognosis factor. Additionally, when G6PD expression was silenced in bladder cancer cells, increased intracellular ROS, suppressed proliferation, and increased apoptosis were observed. These results suggest the potential clinical utility of 6-aminonicotinamide (6-AN), an NADP analog inhibitor of G6PD. In fact, 6-AN has been previously demonstrated to sensitize cancer cells to cisplatin [87]. Emodin (1,3,8-trihydroxy-6-methylanthraquinone), a natural anthraquinone that is found in traditional Chinese herbal medicines, also enhances the chemosensitivity of BCa cells by elevating the levels of intracellular ROS [88]. Treatment strategies focused on enhancing tumor cell susceptibility to chemotherapeutics would prove invaluable clinically, where chemoresistance is extremely prevalent and plagues the effectiveness of current therapeutic approaches.

Cordycepin, an adenosine derivative, was demonstrated to enhance intracellular ROS levels in T24 BCa cells, inducing increased apoptosis that was absent with concurrent administration of the potent antioxidant N-acetylcysteine [89]. Withaferin A, a steroidal lactone, induced DNA strand breaks and apoptosis via ROS production in human BCa cells [90]. Using a subcutaneous xenograft murine model, Duan et al. [91] reported that treatment with vitamin K2, despite its antioxidant properties, inhibited tumor growth and reduced tumor volume via activation of ROS-JNK/p38 apoptotic pathways. Simultaneous administration with N-acetylcysteine led to the attenuation of these anti-cancer effects. Buthionine sulfoximine (BSO) and disulfiram appear to be promising candidates for attenuating the GSH/GPx antioxidant system [92,93]. Clinical trials are currently underway for the use of BSO against neuroblastoma and disulfiram against both breast and pancreatic cancers [78]. Several pharmacological approaches to attenuating the thioredoxin (TRX) antioxidant pathway have also been applied in the field of oncology. PX-12 (1-methylpropyl 2-imidazolyl disulfide) is a small molecule inhibitor of TRX-1 and has been shown to induce apoptosis in mouse osteosarcoma cells and attenuate progression of osteosarcoma in a murine model [94]. A previously identified anti-cancer compound, jolkinolide B, was demonstrated to function by inhibiting thioredoxin reductase 1 as well as depleting GSH levels [95]. In chemoresistant bladder cancer cells, jolkinolide B was shown to induce both apoptosis and paraptosis [95]. While pro-oxidant therapeutic approaches such as these have very promising potential, the induction of ROS production can damage non-cancerous tissue and lead to serious side effects [78].

**Table 2:** Pro-oxidants as anti-bladder cancer agents.

Pro-Oxidant Agent	Model System	Observed Effect	Reference
Emodin (1,3,8-trihydroxy-6-methylanthraquinone)	In vitro human BCa cells	Enhanced susceptibility to cisplatin	[88]
Cordycepin	In vitro human BCa cells	Increased tumor cell apoptotic cell death	[89]
Withaferin A	In vitro human BCa cells	Increased tumor cell DNA damage and apoptotic cell death	[90]
Vitamin K2	Mouse, tumor site injection	Decreased tumor burden and increased tumor cell apoptosis	[91]
Jolkinolide B	In vitro cisplatin resistant-BCa cells	Induced apoptosis and paraptosis	[95]

## 4. Conclusions

The current BCa treatment paradigms have limited therapeutic impact, suggesting a need for investigation of novel therapies. Extensive research has been conducted on the role of ROS in the development and progression of cancer, including bladder cancer. Therapies including antioxidant-targeted therapies have demonstrated promising outcomes both in vitro and in vivo. With elevated ROS being a ubiquitous characteristic of cancer, oxidative stress-based therapeutic approaches extend beyond the scope of bladder cancer alone, and if effective clinically, they could provide a treatment approach that is widely effective despite heterogenicity among tumor types.

## Figures and Tables

**Table 1:** Anti-tumor effects of antioxidants against bladder cancer.

Antioxidant Agent	Model System	Observed Effect	Reference
N-acetylcysteine	In vitro human BCa cells	Depleted cancer cell viability, adhesiveness, migratory abilities, and invasiveness	[71]
Vitamin A (retinol)	Mouse, oral administration	Diminished early BCa progression	[72]
Epigallocatechin gallate (EGCG)	Mouse, IP injection	Decreased tumor burden, downregulated NF-κB and MMP-9	[73]
Ellagic acid	In vitro human BCa cells	Decreased cell proliferation	[74]

BCa, bladder cancer; IP, intraperitoneal; NF-κB, nuclear factor-kappa B; MMP-9, matrix metalloproteinase-9.
